# Congenital Corneal Anesthesia Secondary to Brainstem Ischemia as a Rare Complication of Neonatal Hypoxic-Ischemic Encephalopathy

**DOI:** 10.7759/cureus.87777

**Published:** 2025-07-12

**Authors:** Mohammed Frarchi, Nabil Bouslous, Jalal Laakri, Khaoula Ajbar, Omar Moustaine

**Affiliations:** 1 Ophthalmology, Souss-Massa University Hospital, Agadir, MAR

**Keywords:** brainstem lesions, congenital corneal anesthesia, corneal ulceration, hypoxic ischemic encephalopathy, neurotrophic keratopathy

## Abstract

We report the case of a very rare instance of congenital corneal anesthesia secondary to brainstem lesions following neonatal hypoxic-ischemic encephalopathy. It’s the case of a 7-month-old baby with a history of neonatal asphyxia who presented with spontaneous corneal ulcerations and corneal anesthesia associated with brainstem lesions and brain lesions visible on MRI, secondary to neonatal hypoxic-ischemic encephalopathy. The case of the baby was complicated by the superinfection of the ulceration. He was treated with fortified antibiotics, eyedrops, and frequent instillation of eye lubricants and ointments. The evolution was marked by the healing of the ulceration and the improvement of the corneal sensitivity; however, there were sequelae in the more affected eye by the formation of a corneal pannus.

## Introduction

Congenital corneal anesthesia (CCA) is a rare condition characterized by the lack of corneal sensitivity. The cornea’s rich sensory innervation plays a crucial role in maintaining ocular surface homeostasis. The disruption of this sensory system predisposes the ocular surface to recurrent ulceration that can evolve into a neurotrophic keratopathy and all the complications that accompany it [1].

Neonatal hypoxic-ischemic encephalopathy (HIE) is a severe perinatal condition caused by reduced oxygen and blood flow to the brain, often leading to neurological injury. Brainstem damage in HIE has been described in previous reports; however, it still lacks detailed descriptions of clinical and radiological findings, since most patients with brainstem lesions die during delivery or a few days after birth [2,3]. In this case report, we describe a rare instance of CCA secondary to ischemic brainstem lesions following neonatal HIE.

## Case presentation

A seven-month-old boy was presented to our ophthalmology department with a red left eye associated with eyelid swelling. Medical history revealed that he was born at term, following a third pregnancy for his mother, with a birth weight of four kg (normal weight at birth). His delivery was complicated by prolonged labor, resulting in neonatal asphyxia that necessitated a week’s hospitalization in the neonatal intensive care unit. At six months of age, he started experiencing epileptic seizures, for which he is currently receiving treatment.

When examining the patient, the general examination revealed a hypotonic baby.

**Figure 1:**
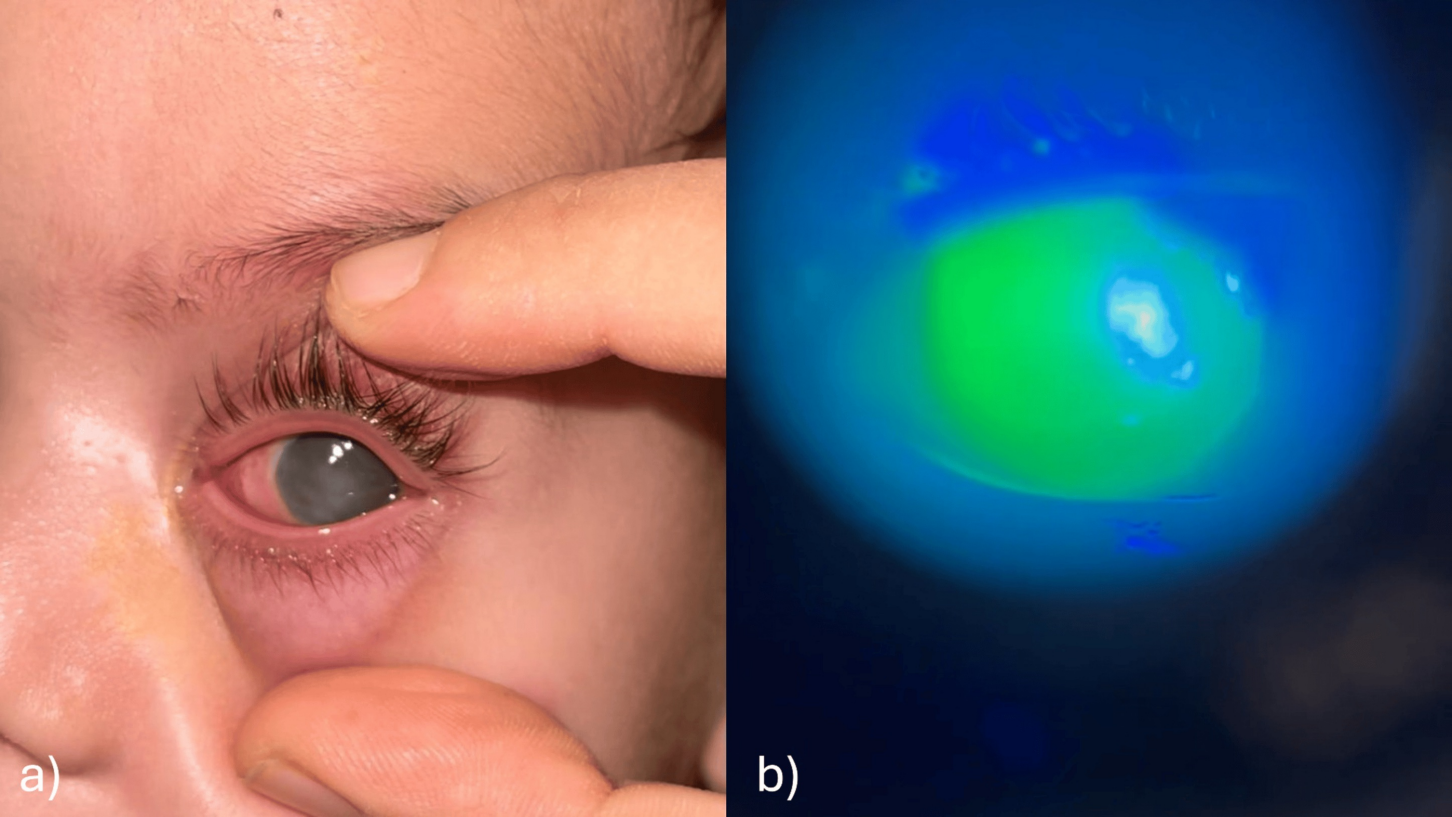
Images at admission showing a corneal edema of the left eye with a discrete central opacification (a), and a corneo-conjunctival ulcer after fluorescein instillation (b).

Ophthalmologic examination revealed a normal right eye, while the left eye showed a corneal edema and corneo-conjunctival ulcer with a discrete whitish opacity (Figure 1).

The mother denied any history of trauma or exposure to irritants or chemicals. The baby was admitted and treated with topical antibiotics (tobramycin eye drops five times a day), artificial tears, and vitamin A ointment. The ulceration improved significantly, achieving complete re-epithelialization. Suspecting a vitamin A deficiency was the cause, vitamin A levels were requested, and the patient was discharged pending results.

**Figure 2:**
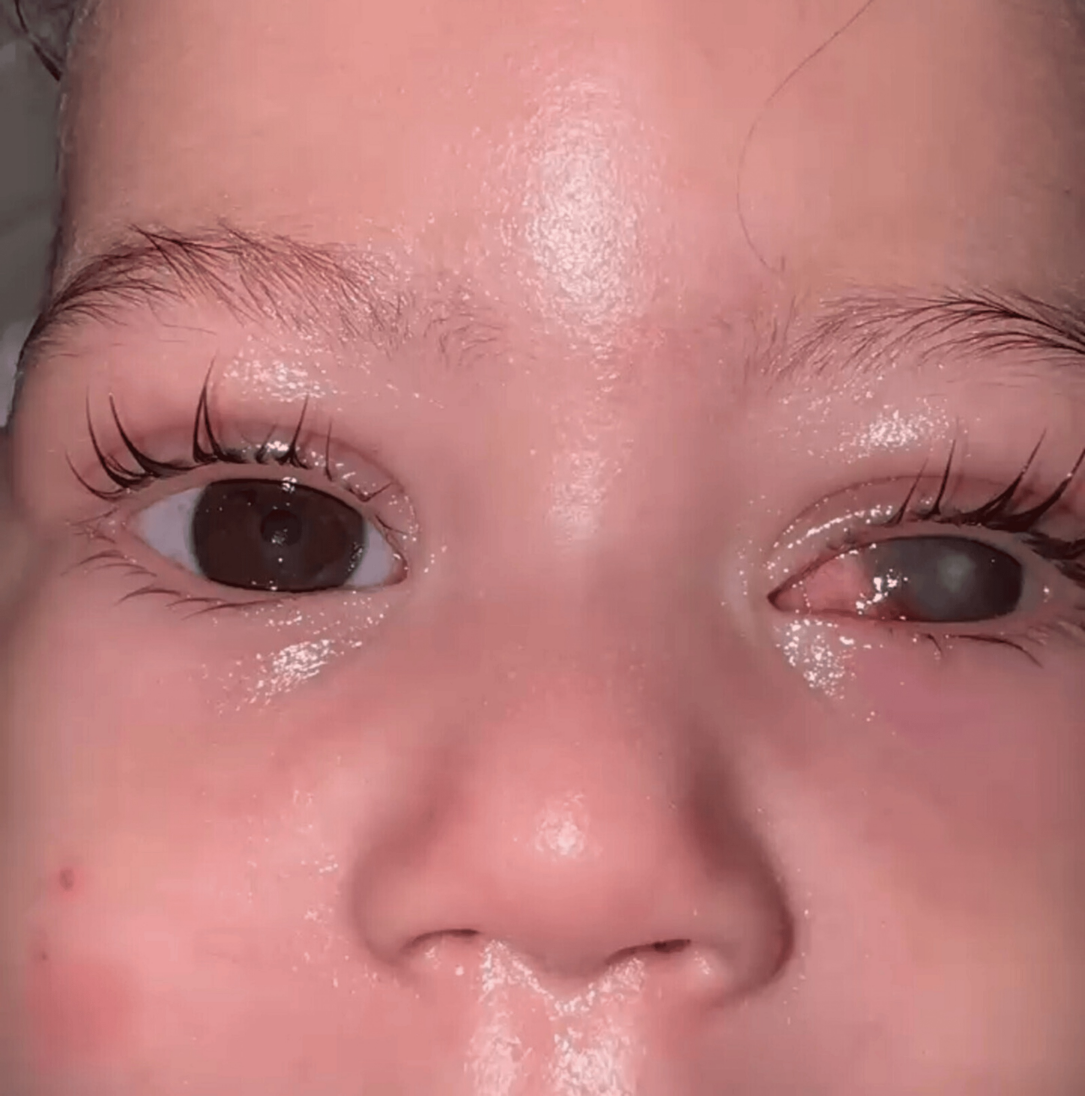
Image at readmission showing a corneal ulcer of the right eye, a superinfected corneal ulcer of the left eye, and a calm, unbothered baby.

Five days later, the baby returned with bilateral corneal edema and ulceration and superinfection of the ulcer in the left eye (Figure 2). Upon further questioning, it was revealed that the mother had discontinued the prescribed treatment after discharge.

**Figure 3:**
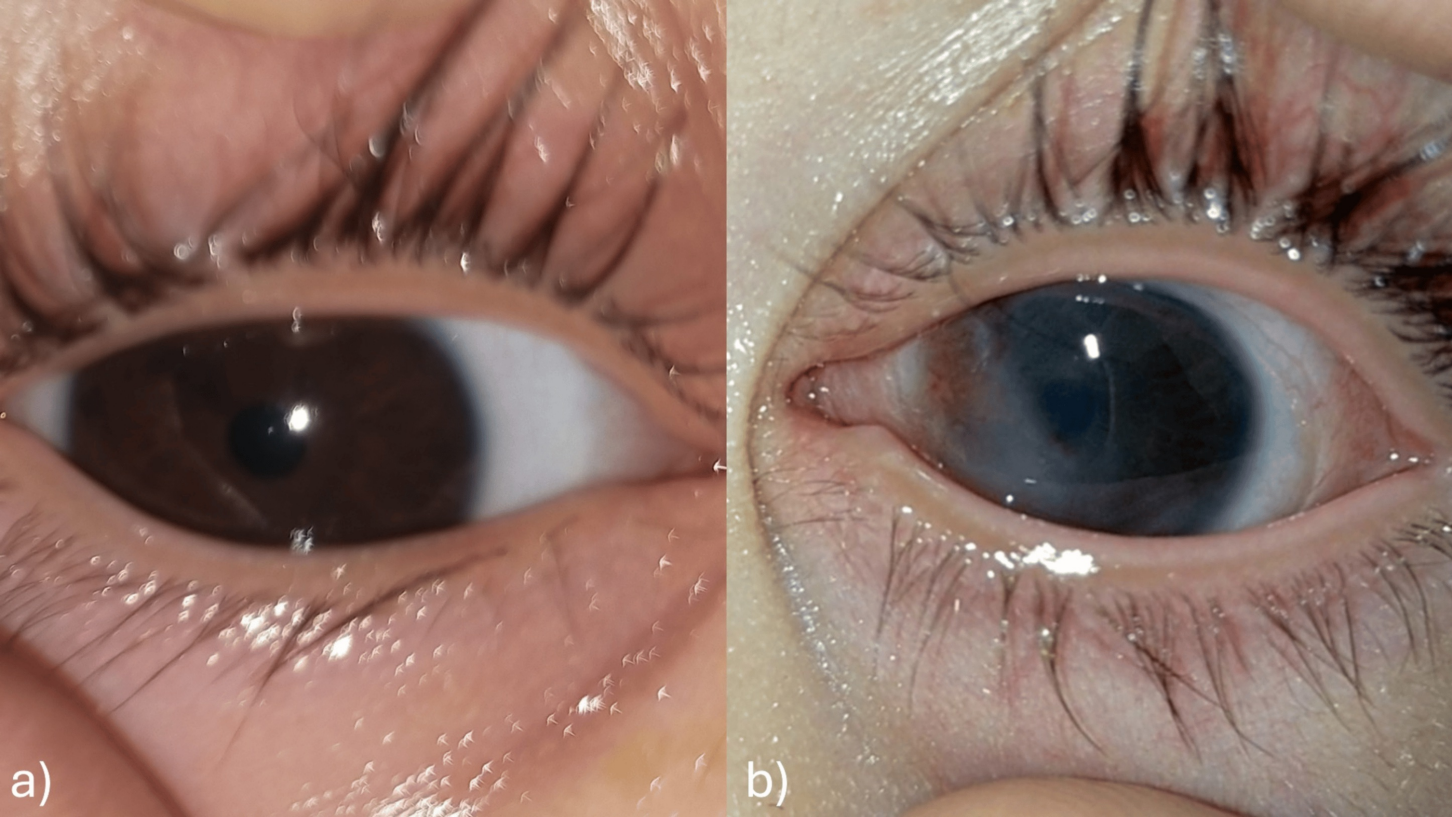
mages at discharge after the first readmission showing complete healing of the right eye (a), and persistence of nasal corneal opacity and neovascularization in the left eye (b).

The patient was readmitted, undergone corneal scraping and was put on broad-spectrum topical fortified antibiotics eyedrops (vancomycin “50 mg/ml” + ceftazidime “50 mg/ml” hourly drops for 48 H then eight times a day), artificial tears (eight times a day), and vitamin A ointment (two times a day). The corneal scraping turned out negative. The evolution was marked by the complete re-epithelialization of the right eye without scarring, while the left eye re-epithelialized with the persistence of nasal and central corneal scarring and neovascularization (Figure 3). The mother was counseled on the importance of strict adherence to treatment, particularly the use of artificial tears.

**Figure 4:**
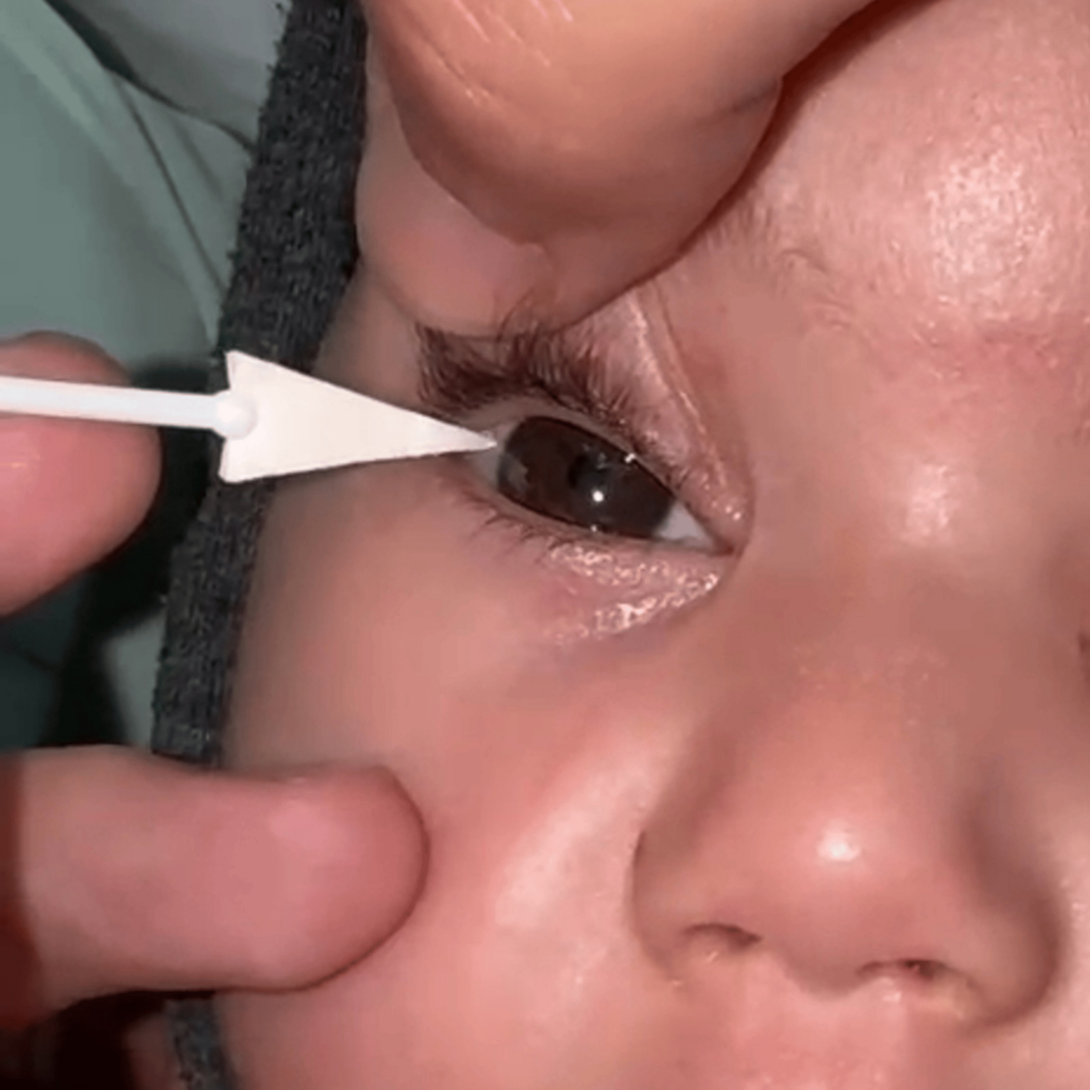
Corneal sensitivity testing showing poor reaction of the infant to the cotton wisp test.

Vitamin A levels returned within normal limits, ruling out deficiency. Further evaluation revealed corneal anesthesia and reduced facial sensitivity, prompting a suspicion of congenital corneal anesthesia (Figure 4).

**Figure 5:**
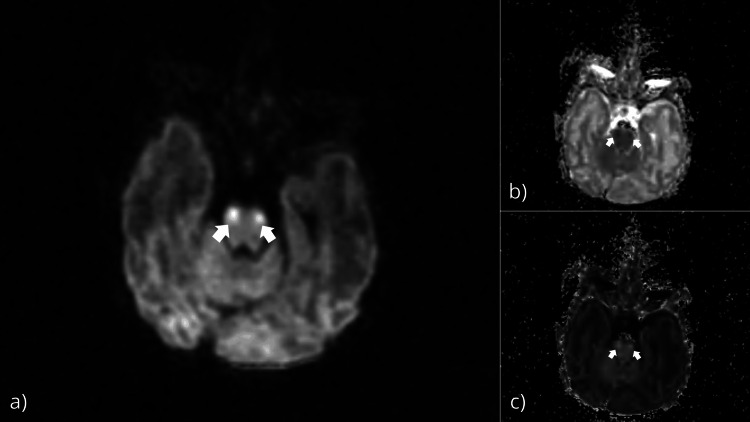
Images of MRI performed at 1 month of age showing ischemic lesions of the brainstem (white arrows) in Axial DWI (a), ADC (b), and e-ADC (c). DWI: diffusion-weighted imaging; ADC: apparent diffusion coefficient

Given the history of perinatal asphyxia, neurological origin was suspected. Reassessment of an MRI performed at one month of age that stated initially the presence of “bilateral and asymmetrical periventricular cystic lesions, suggestive of sequelae to the perinatal asphyxia, along with signal abnormalities in the splenium of the corpus callosum” has identified localized ischemic lesions in the brainstem that were overlooked in the initial interpretation of the MRI (Figure 5). The localization of the ischemic lesions in the pons, which is where the trigeminal nuclei are located, is consistent with the trigeminal nerve’s nuclei involvement and explains the corneal anesthesia. Examination of the rest of the cranial nerves showed a normal photo-motor reflex, normal oculomotricity, normal auditory evoked potential, and good suction. However, the rooting reflex was absent, which, in addition to the epileptic seizures, is also an indication of brainstem malfunction.

**Figure 6:**
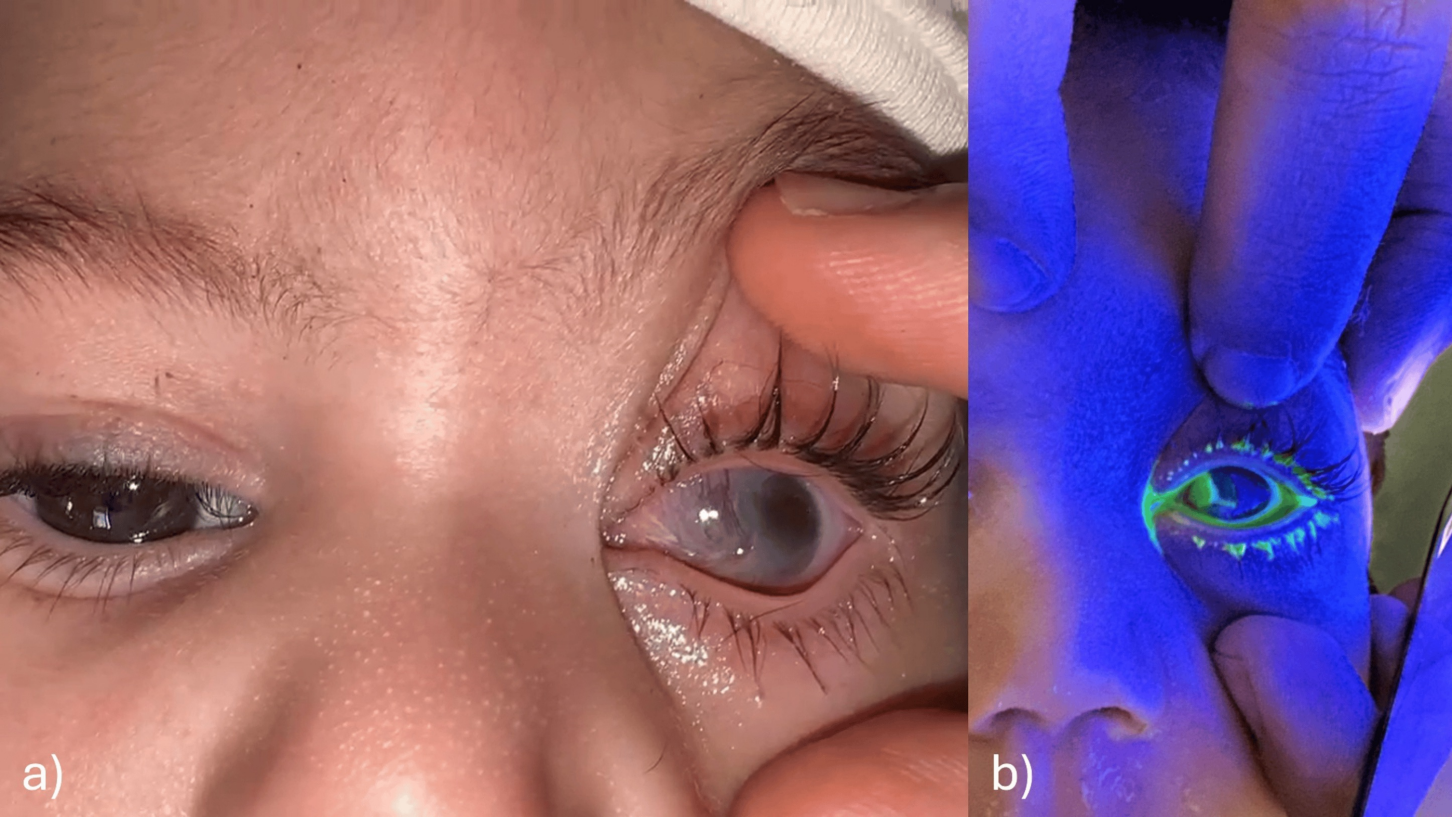
Images at the second readmission showing corneal edema (a) and fluorescein uptake (b) in the left eye.

The baby was discharged and kept permanently under artificial tears every two hours and carbomer ointment at night and was followed up every 48h for a week, then every week for a month, then every two weeks. During the first month and a half of the follow-ups, there was no recurrence of the ulceration with the beginning of formation of a corneal pannus in the left eye (The more severely affected). After this period, there was a recurrence of the ulceration in the left eye with superinfection of the ulcer (Figure 6) for which he was readmitted and treated with fortified antibiotic eyedrops following the same previous protocol.

The infection was most probably due to the use of the eye drops and ointment that were kept in unhygienic conditions (The medications were kept in a plastic bag in which there was leakage of the ointment from a perforated ointment tube). After management of the infection and the resolution of the ulceration, leaving a more advanced corneal pannus. The mother was well instructed about the importance of keeping the medication in the most hygienic conditions to prevent such recurrences.

**Figure 7:**
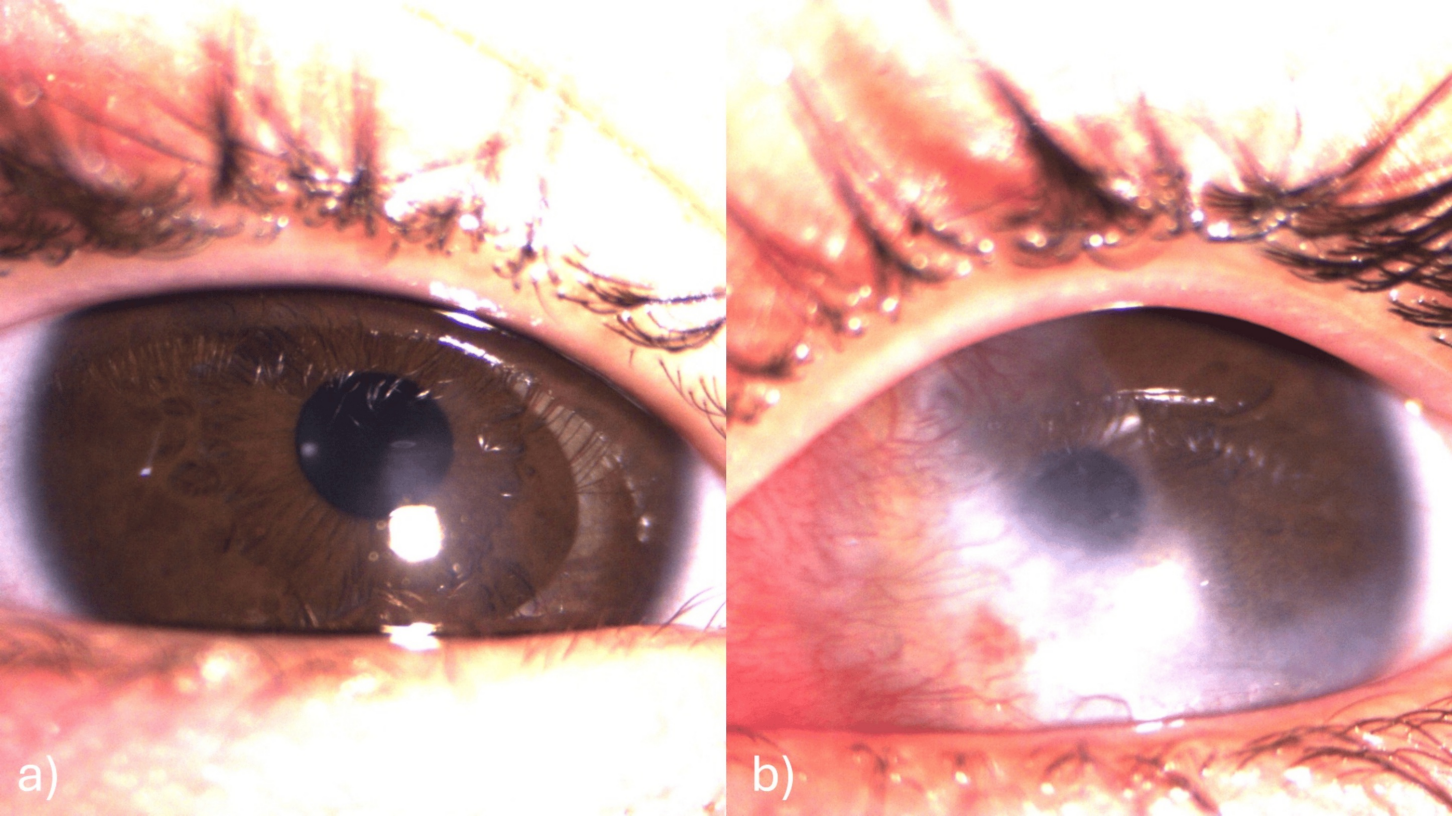
Images at final discharge of the baby: Right eye (a) and left eye (b).

The follow-ups during 3 months after the last hospitalization were all good with no signs of recurrence and with the improvement of corneal sensitivity; however, there was significant corneal pannus in the left eye (Figure 7).

## Discussion

The corneal sensation has a big role in maintaining the trophicity and the integrity of the corneal epithelium through the blink reflex and the tearing reflex; it also enhances epithelial cell proliferation by neurotransmitters and nerve growth factors released from corneal nerve endings [1,4]. Thus, any malfunction of this sensation puts the ocular surface at risk of injury in addition to a delay in the healing and regeneration process.

Congenital trigeminal anesthesia is a rare entity characterized by neurosensory deficits that involve the ocular surface and may extend to other divisions of the trigeminal nerve [5]. The deficit is usually bilateral; however, some unilateral cases have been reported [6,7].

CCA can be isolated or take part in a complex neurological syndrome. The clinical diagnosis is usually challenging, and the time of the diagnosis can go from a few months to a few years after birth, but usually it’s between 8 months and 12 months [5]. The frequency of patients diagnosed at this particular age is thought to be due to the change of sleeping habits of infants, as they get older and require less sleep, the eyes are exposed to the air for much greater periods of time, and thus a greater risk of injury [8]. The diagnosis should be suspected when facing a child with corneal erosions without any discomfort, reduced reflex tearing and blink rates, impassive response to eyedrop instillation, or increased mucus secretions [5]. The diagnosis can be confirmed by a cotton wisp test, and corneal anesthesia can be accurately assessed with an esthesiometer that can give us an idea about the severity of the disease [1]. The initial presentation can be of an asymptomatic infant with only reduced blinking and lacrimation or as mentioned before in our case report in which the presentation was in the form of corneal ulceration and congested conjunctiva either unilaterally or bilaterally with an unusual calm child, and sometimes in advanced stages the presentation of the disease can be in the form of corneal opacities due to repeated corneal ulcerations.

The progression of these neurotrophic changes were graded into three stages by Mackie [9]: stage one: usually a punctate keratopathy; stage two: persistent epithelial defect with smooth and rolled edges associated with Descemet’s membrane folds and stromal swelling which was the case of our patient; and finally the stage three: corneal stromal melting and lysis that can lead to perforation.

Rosenberg classified Congenital trigeminal anesthesia (CTA) cases into three groups [8]: the first group are patients with isolated CTA with no evidence of other cranial nerve disfunction or mesenchymal tissue abnormalities, the second group consists of patients who had CTA associated with multiple developmental anomalies and the third group is made up of patients with CTA associated with neurological features indicating focal intrinsic brainstem dysfunction with no evidence of associated somatic structural abnormalities [8].

Our patient can be classified in the third group of Rosenberg’s classification, with the presence of CCA associated with brainstem lesions visible on MRI.

Lesions of the brainstem have been reported in cases of HIE at post-mortem examination by Leech and Alvord and on MR images by Sugama and Eto in a case series of children [2,10]. The MR imaging in these cases shows symmetric bilateral lesions of the brainstem that have a faintly hyperintense signal on T2 [3]. In our case, the lesions were visible on the MRI as symmetric hyperintense bilateral lesions on the diffusion images and the e-ADC and hypointense on the ADC images, which can be translated into poor tissue diffusion in correlation with poor perfusion or ischemic lesions of the brainstem.

Many clinico-radiological manifestations of brainstem injury secondary to HIE have been reported in the literature, such as oculomotor disturbances, bilateral facial nerve palsy, ventilatory disturbances, and impaired sucking and swallowing [3]. However, cases of damage to the territory of the trigeminal nerve are very rare and have only been reported in two articles by Rosenberg and Hashmi et al. [8,11].

Our case report showcases a very rare condition, which, to our knowledge, has never been published before, of CTA secondary to brainstem lesions as a complication of HIE, with clear MR imaging of the brainstem lesions.

Management of these cases must start by educating the parents about the disease and giving them information about avoiding or taking extra caution in situations where corneal injury is more likely to happen (bad weather, bath-time) and to seek urgent medical attention if a corneal ulcer is suspected (red eye, corneal opacity, ocular purulent secretions) [12]. This is very important as improper knowledge of the parents can complicate the management of these already delicate cases, and a prime example of it is in our case, when the mother stopped her son’s treatment after being discharged from the hospital. The medical management of these cases relies on frequent instillation of preservative-free artificial tears and lubricants in addition to antibiotics in case of ulceration. This was sufficient in our case to heal the corneal ulceration. Sometimes surgical treatments are needed, including punctal plugs, temporary tarsorrhaphy, and amniotic membrane grafting [12]. Corneal grafting has a poor outcome in these cases due to the poor healing process of these patients, who usually develop allograft rejection [5].

## Conclusions

CCA is a rare entity, the diagnosis of which can be challenging. The early diagnosis and management of this condition prevent severe morbidity that can go as far as vision loss, which prompts the need for more awareness of this disease by ophthalmologists and pediatricians.
